# Modulation of *Pleurodeles waltl* DNA Polymerase mu Expression by Extreme Conditions Encountered during Spaceflight

**DOI:** 10.1371/journal.pone.0069647

**Published:** 2013-07-31

**Authors:** Véronique Schenten, Nathan Guéguinou, Sarah Baatout, Jean-Pol Frippiat

**Affiliations:** 1 Stress Immunity Pathogens Laboratory, EA7300, Lorraine University, Vandœuvre-lès-Nancy, France; 2 Radiobiology Unit, Belgian Nuclear Research Centre, SCK•CEN, Mol, Belgium; 3 Department of Molecular Biotechnology, Ghent University, Ghent, Belgium; Louisiana State University and A & M College, United States of America

## Abstract

DNA polymerase µ is involved in DNA repair, V(D)J recombination and likely somatic hypermutation of immunoglobulin genes. Our previous studies demonstrated that spaceflight conditions affect immunoglobulin gene expression and somatic hypermutation frequency. Consequently, we questioned whether Polμ expression could also be affected. To address this question, we characterized Polμ of the Iberian ribbed newt *Pleurodeles waltl* and exposed embryos of that species to spaceflight conditions or to environmental modifications corresponding to those encountered in the International Space Station. We noted a robust expression of Polμ mRNA during early ontogenesis and in the testis, suggesting that Polμ is involved in genomic stability. Full-length Polμ transcripts are 8–9 times more abundant in *P. waltl* than in humans and mice, thereby providing an explanation for the somatic hypermutation predilection of G and C bases in amphibians. Polμ transcription decreases after 10 days of development in space and radiation seem primarily involved in this down-regulation. However, space radiation, alone or in combination with a perturbation of the circadian rhythm, did not affect Polμ protein levels and did not induce protein oxidation, showing the limited impact of radiation encountered during a 10-day stay in the International Space Station.

## Introduction

The immune system is affected by environmental changes encountered during spaceflight such as confinement, microgravity, biomechanical stresses, radiation and disruption of the circadian rhythm. Indeed, astronauts have experienced altered immune function and increased vulnerability to infections during spaceflights dating back to the Apollo and Skylab missions [1]. The effects of spaceflights on the immune system should therefore be considered more thoroughly before undertaking prolonged space missions.

Until now, studies regarding spaceflight-induced immune dysfunctions focused mostly on innate immunity and T-cell responses while humoral immunity and antibodies, despite their important functions, have rarely been investigated [2]. To improve our knowledge concerning the effects of spaceflight on humoral immunity, we used the Iberian ribbed newt *Pleurodeles waltl* as animal model. This amphibian species is a good model for studies applicable for human health because it was shown that the cardinal elements of the adaptive immune system are shared by all gnathostomes [3]. This observation was confirmed in *P. waltl*. Indeed, it was shown that this animal possesses at least 10 subgroups of VH genes, 8 JH and 8 D segments in its genome and that it uses these genes and segments in the same way as humans and rodents to create its functional antibody heavy chain genes [4]. It was also demonstrated that *P. waltl* possesses three isotypes of antibodies, IgM, IgY and IgP, which have their human physiological counterparts [5]. And finally, several immunologically important genes and transcripts, such as those encoding AID, Ikaros and the C3 complement component, were shown to be conserved in *P. waltl* and have expression patterns similar to those observed in mammals ([6], [7] and unpublished data).

Using that species, we previously showed that spaceflight affects antibody production in response to an antigenic stimulation. Indeed, the expression of IgY heavy chains (the physiological counterpart of mammalian IgA molecules) was increased in flown *P. waltl* [8] as previously observed in cosmonauts [9]. Furthermore, the use of the different VH gene subgroups [8] and the expression of individual VH genes [10] were observed to be modified under spaceflight conditions. In addition, we recently demonstrated that somatic hypermutation, that diversify antibody binding-sites to improve their affinity, occur in space following immunization but at a lower frequency [11] and that gravity changes during animal development affect IgM heavy chain transcription [12].

To create functional immunoglobulin (Ig) genes, B lymphocytes from jawed vertebrates associate V_H_-D, D-J_H_, V_κ_-J_κ_ and V_λ_-J_λ_ gene and segments during their development in primary lymphoid organs. This process, called V(D)J recombination, involves DNA double-strand breaks inflicted by the products of the RAG1 and RAG2 recombination-activating genes [13]. The processing of V(D)J coding joints is then sustained by core NHEJ components (Ku-Artemis-DNA-PK and XLF-XRCC4-DNA ligase IV) [14] but DNA polymerases of the X family further contribute, particularly when the generated nicks leave 3′ overhangs, which are good substrates for the polymerases [15]–[17]. The polymerases of the X family include the DNA polymerases beta (Polβ), lambda (Polλ), mu (Polμ) and terminal deoxynucleotidyl transferase (TdT), which are structurally related enzymes specialized in repair pathways involving double-strand breaks (DSBs) and gaps [18]. Polµ is more closely related to TdT than to other polymerase X members and shares the same exon-intron organization. However, the roles of TdT and Polµ are distinct [19].

In 2003, Bertocci et al. reported that Ig light chain gene rearrangement is altered in Polµ-deficient mice, with an extensive reduction in κ light chain CDR3 length [20]. This observation highlighted the contribution of this protein in Ig light gene rearrangement, in contrast to TdT, which is mainly involved in Ig heavy chain gene rearrangement [21], [22]. This difference is reinforced by the fact that the Polµ and TdT expression patterns are different. In adults, Polµ is expressed in a wide range of tissues [23], while TdT expression is limited to hematopoietic organs.

Several studies suggested that Polµ could also be involved in somatic hypermutation (SHM) of Ig genes that diversify antibody binding sites [24]–[27].

Finally, it was shown that Polµ is involved in the repair of radiation-induced DNA damage. Indeed, exposure of cells to ionizing radiation (IR) induced a reduction in properly spliced Polµ mRNAs [23] and an increase in Polµ protein that forms discrete nuclear foci coinciding with IR-induced foci of γH2AX, a marker of sites of DNA DSBs [28]. Another study showed that a lack of Polµ results in delayed DSB repair kinetics and in persistent DNA damage [29].

As these data indicate that Polµ is involved in V(D)J recombination, DNA repair and likely somatic hypermutation, we characterized that polymerase in *P. waltl* and determined how spaceflight-associated environmental modifications affect Polµ expression during *P. waltl* development in the International Space Station. This study is important because several studies showed that spaceflight has a negative impact on the immune system (see above) and exposure of astronauts to low doses of cosmic radiation is considered as a threat for long-term space missions [30].

## Materials and Methods

### Ethic Statement

Animals were treated in accordance with the National Legislation and the Council Directive of the European Communities on the Protection of Animals Used for Experimental and Other Scientific Purposes (86/609/EEC). Animal husbandry conditions were approved by the French Ministry of Agriculture and Fisheries (agreement DDSV54/SPA/07/130) and the protocol was approved by the regional ethic committee: Comité régional d’éthique pour l’expérimentation animale, Nancy - Lorraine - Nord-Est (Permit Number: 04112004).

### Animals and RNA Extractions

*P. waltl* adults and embryos were reared in our animal facilities in running tap water at 18°C. Embryonic and larval developmental stages were defined according to Gallien and Durocher [31]. Ten embryos were randomly chosen for each developmental stage studied and homogenized in TRIzol reagent (Invitrogen, Carlsbad, CA, USA) for RNA extraction. Total RNA was also isolated from the thymus of stage 44 (36-day-old) larvae and from various adult tissues.

### AMPHIBODY Space Experiment

This experiment was performed in the International Space Station (ISS) from March 30 to April 09, 2006 using miniaquaria developed by EADS-Astrium and the German Space Agency [12]. Six miniaquaria were mounted on the stationary positions of the Kubik incubator designed for space experiments (Comat Aerospace, Toulouse, France) for microgravity exposure, 6 on incubator’s 1 g-centrifuge to perform an in-flight 1 g control and 12 in an incubator kept on Earth as ground controls. Embryos developed during 10 days in the ISS. Living larvae were recovered and fixed 14 h after landing. Note that the Kubik’s centrifuge was unexpectedly shut off 30 h before landing, thereby exposing larvae to several hours of microgravity and invalidating our in-flight 1 g control.

### Other Animal Treatments

*P. waltl* embryos exposures to *i*) hypergravity in a centrifuge running at 3G, *ii*) simulated microgravity in a random positioning machine (RPM) that randomize the direction of the gravity force resulting in an average net force approaching zero, *iii*) altered circadian rhythm and *iv*) simulated ISS radiation environment using a combination of neutrons (total dose of 2.0 mGy) and γ rays (total dose of 1.9 mGy) as representative of low-linear energy transfer (LET) and high-LET particles received during 10 days in the space station, were performed as previously described [12]. Note that a full reproducibility of space radiation on Earth is not possible this is why we use the word ‘simulation’. Additionally, to induce a mechanical stress, *P. waltl* larvae at stage 36–37 of development were placed in 50 mL of water at 20°C and subjected to vibrations for 5 h at 15 Hz. In each case, larvae of the same developmental stage reared under standard conditions were used as controls.

### Cloning and Sequencing of *P. waltl* Polymerase µ cDNA

One microgram of spleen RNA was reverse transcribed using random primers and M-MLV reverse transcriptase (Invitrogen, Carlsbad, CA, USA). Primers were designed in conserved regions identified by aligning Polµ mRNA sequences from *Homo sapiens*, *Mus musculus*, *Danio rerio* and *Xenopus tropicalis*. A first PCR reaction was performed with the Polµ For and Polµ Rev primers (Table 1) on spleen cDNA using Goldstar Taq polymerase (Eurogentec, Seraing, Belgium). Two PCR products of 917 and 814 bp were obtained, cloned and sequenced. Next, specific primers (Polmu GSP1 and Polmu GSP2) and nested primers (Polmu NGSP1 and Polmu NGSP2, Table 1) were designed from the obtained sequence to perform 3′- and 5′-RACE using the BD SMART RACE cDNA Amplification kit (Clontech, Mountain View, USA). RACE products were cloned and sequenced to obtain full-length *P. waltl* Polµ cDNA.

**Table 1:** Primers used in this study.

Primer name	Primer sequence (5′ to 3′)	Purpose	Annealing temp. (°C)
Polmu For	CCAGTATATGCCTGCCAGAGA	PCR	63
Polmu Rev	TGCTTTGATCCAGTCCACCC	PCR	63
Polmu GSP1	ACTGTTGCCGAACTCTGCCTCCTCT	5′-RACE	63
Polmu NGSP1	GCCAGTATCTCCAAAGCATCCGTGA	5′-RACE	63
Polmu GSP2	AGAGCTCCAGCACAGCAGCAGAAAA	3′-RACE	64
Polmu NGSP2	CATTTGCTCTGCTTGGGTGGACTG	3′-RACE	64
Polmu long For	TGGAAGCTGACAAGATCCAG	qPCR & PCR	59
Polmu long Rev	CTGCTTCCCTCTTTGAAACC	qPCR	59

### Real-time PCR

qPCR primers were designed using the GenScript software. Polμ primers (Polmu long For and Polmu long Rev) were defined in exons 7 and 8 to quantify only the longest isoform (Table 1). DNA-PKc-, Gadd45-, GAPDH-, α-actin-, TAFII- and mtRNA16S-specific primers were described previously [12]. qPCRs were performed using the Mesa Green qPCR Master Mix (Eurogentec, Seraing, Belgium) in a Mastercycler® realplex2 real-time PCR engine (Eppendorf, Hamburg, Germany). The cycling protocol was as follows: 3 min at 95°C, then 40 cycles of 15 s at 95°C and 30 s at 59°C. Standard curves were generated to ensure that the amplification efficiencies were similar and in the range of 95%–105%. Each qPCR was performed in triplicate and repeated at least 2 times. The data were analyzed using the relative Pfaffl model [32]. Relative expressions, expressed in arbitrary units (A.U.), were calculated using 4 housekeeping genes (α-actin, mtRNA16S, TafII and GAPDH), the GeNorm software and the Vandesompele method [33]. Primers Polmu long For and Polmu Rev (Table 1) were used to amplify, using conventional PCR, Polµ variants in the spleen and testis of *P. waltl*.

### Production of Anti-polymerase µ Polyclonal Antibodies

To produce polyclonal antibodies specific for *P. waltl* Polµ, a 16 amino acid peptide was designed, produced and coupled to KLH by Eurogentec (Seraing, Belgium). BALB/c mice were immunized with this immunogen. The first dose consisted of 100 µg of the immunogen emulsified with TiterMax® adjuvant (Sigma-Aldrich, Saint-Quentin Fallavier, France) in a 1∶1 ratio. Three weeks later, a second dose of 100 µg of immunogen mixed with TiterMax® in a 1∶0.5 ratio was injected. One week after this second injection, mice antisera were collected, purified with the Rapid Antibody Purification Kit (Cell Biolabs, San Diego, CA, USA) and concentrated using a Centricon Plus-70 device (Millipore, Billerica, MA, USA).

### Western Blotting

Proteins were prepared by lysing a pool of 3 larvae in lysis buffer containing Triton X-100. Samples of 50 µg of protein were heated at 97°C for 5 minutes, run on a 12% Bis-Tris gel and electrotransferred to a PVDF membrane (Amersham, Buckinghamshire, UK). Immunodetection was performed using homemade polyclonal antibodies against Polµ (see above) and a horseradish peroxidase (HRP)-conjugated rat anti-mouse secondary antibody. Immunodetection was performed using the SuperSignal West Pico Chemiluminescent Substrate (Pierce, Erembodegem, Belgium).

### Protein Oxidation

The detection of oxidatively damaged proteins was performed with an OxiSelect™ Protein Carbonyl Immunoblot Kit (Cell Biolabs, Inc., San Diego, CA, USA) according to the manufacturer’s recommendations. Briefly, proteins were separated by SDS-PAGE and blotted on PVDF membranes that were immunostained to detect carbonyls. Carbonyls are primarily aldehydes resulting from oxidation of various amino acids. This staining procedure involves a derivatization of carbonyl groups with 2,4-dinitrophenylhydrazine (DNPH), followed by the detection of DNPH by a specific antibody. Following the oxiblot, membranes were Coomassie Blue stained to ensure equal protein loading in each lane. All measurements of oxidation were normalized to the amount of protein in each sample. To ensure that this approach recognizes only carbonylated proteins, the molecular weight marker, made of recombinant and thus unmodified proteins, was used as a negative control. In all cases, the marker was detected by Coomassie blue staining but not in the oxiblots thereby demonstrating that this protocol reveals only derivatized carbonyl groups and not native proteins.

### Statistics

Statistical analyses were performed using SPSS v13.0 (SPSS Inc., USA). Outlier values were determined by a boxplot of each studied group. When normality and homogeneity of variances were ascertained, as determined by Kolmogorov-Smirnov and Levene tests, respectively, Student's *t*-test analyses were performed to establish two group comparisons. Otherwise, Mann-Whitney tests were used for two group comparisons. p-values <0.05 and <0.10 were selected to indicate significance and trends, respectively.

## Results

### *P. waltl* Polymerase µ

To isolate the Polµ cDNA of *P. waltl*, we designed primers in conserved regions revealed by the alignment of Polµ sequences from different species. These primers allowed the amplification of two products that presented strong homologies with the corresponding regions of human, mouse, Xenopus and zebrafish Polµ mRNAs. Specific primers were then designed to perform RACE-PCR and recover both extremities of *P. waltl* Polµ cDNAs. The sequence of the full-length cDNA has been submitted to the EMBL database (accession number HE583591).

**Figure 1:**
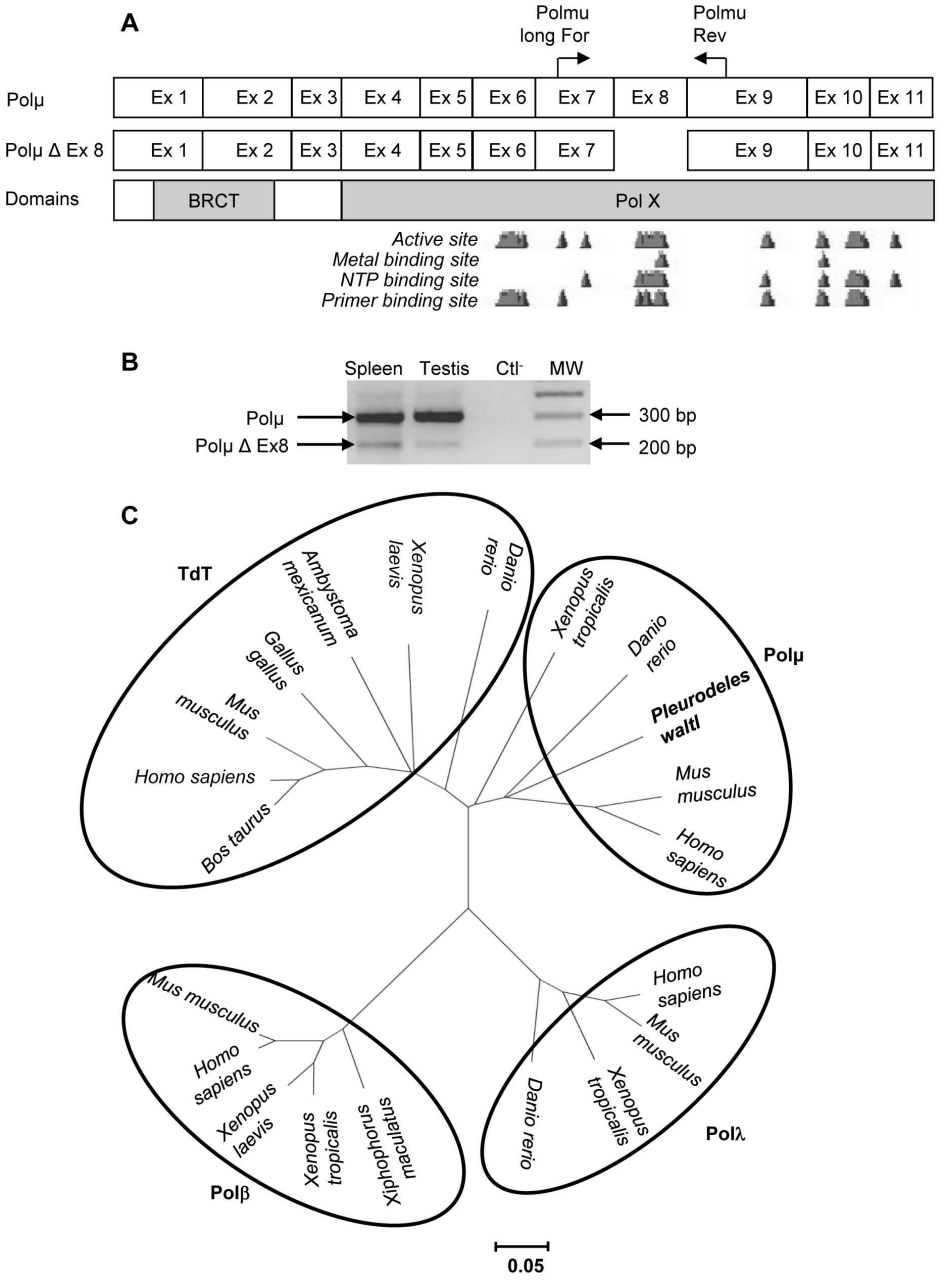
*Pleurodeles waltl* DNA polymerase µ. Schematic representation of *P. waltl* Polµ mRNAs (**A**). The short variant (Polµ Δ Ex 8) presents a 103 bp deletion corresponding to exon 8. This deletion removes one part of the active site of the protein. The BRCT (breast cancer suppressor protein carboxy-terminal) and the Pol X domain, which contains the active site, are required for Polµ function. Polµ variants were amplified using primers indicated by horizontal arrows (**B**). Quantification of the obtained PCR products using a Gel Doc 2000 and the Quantity One v.4.3.1 software (Bio-Rad, Hercules, CA, USA) indicates that the short isoform represents 16% and 6% of the Polµ transcripts in the spleen and testis of *P. waltl*, respectively. Ctl^−^ = negative control. MW = 100 bp DNA ladder. A cladogram was then constructed by neighbor joining (**C**) supported with 1000 bootstrap replications using the MEGA4 software (http://www.megasoftware.net/) and the following sequences: Terminal deoxynucleotidyl transferase (TdT); *Bos taurus* (DAA14763), *Homo sapiens* (BAB72001), *Mus musculus* (NP_001036693), *Gallus gallus* (NP_990720), *Ambystoma mexicanum* (AA092254), *Xenopus laevis* (NP_001079251), *Danio rerio* (AAS89780). Polymerase beta; *Mus musculus* (NP_035260), *Homo sapiens* (NP_002681), *Xenopus laevis* (NP_001081643), *Xenopus tropicalis* (AAH74537), *Xiphophorus maculatus* (AAU11319). Polymerase lambda; *Danio rerio* (NP_998408), *Xenopus tropicalis* (NP_001093716), *Mus musculus* (NP_064416), *Homo sapiens* (NP_001167555). Polymerase mu; *Homo sapiens* (NP_037416), *Mus musculus* (NP_059097), *Danio rerio* (NP_956542), *Xenopus tropicalis* (NP_001164987), *Pleurodeles waltl* (HE583591). The scale bar corresponds to the evolutionary distance.

We predicted the *P. waltl* Polµ exon limits by alignment with Polµ cDNA sequences from species whose exon borders are known. This analysis revealed that, as in other species [23], [25], [34], *P. waltl* Polµ is encoded by 11 exons, of which exon 8 could be alternatively spliced (Figure 1).

To confirm that we isolated the Polµ cDNA of *P. waltl*, we built a cladogram (Figure 1C). This cladogram shows that the sequence identified in *P. waltl* clusters with Polµ sequences from other species, thereby demonstrating that we isolated the cDNA coding for Polµ and not for another polymerase. Moreover, this tree confirms that Polµ is more closely related to TdT than to other polymerase X members and that the divergence of Polµ and TdT is likely much more recent than the divergence of Polβ and Polλ.

Moreover, the two regions known to be necessary for Polµ function are conserved (data not shown): BRCT required for the interaction with core NHEJ factors Ku and XRCC4-Ligase IV [17], [28] and the Pol X domain, which contains the active site (DNA, NTP and metal binding sites) responsible for polymerase activity. Thus, *P. waltl* Polµ has the domains shown to be required for its activity in mammals.

### Expression during Early Development and in Adult Tissues

**Figure 2:**
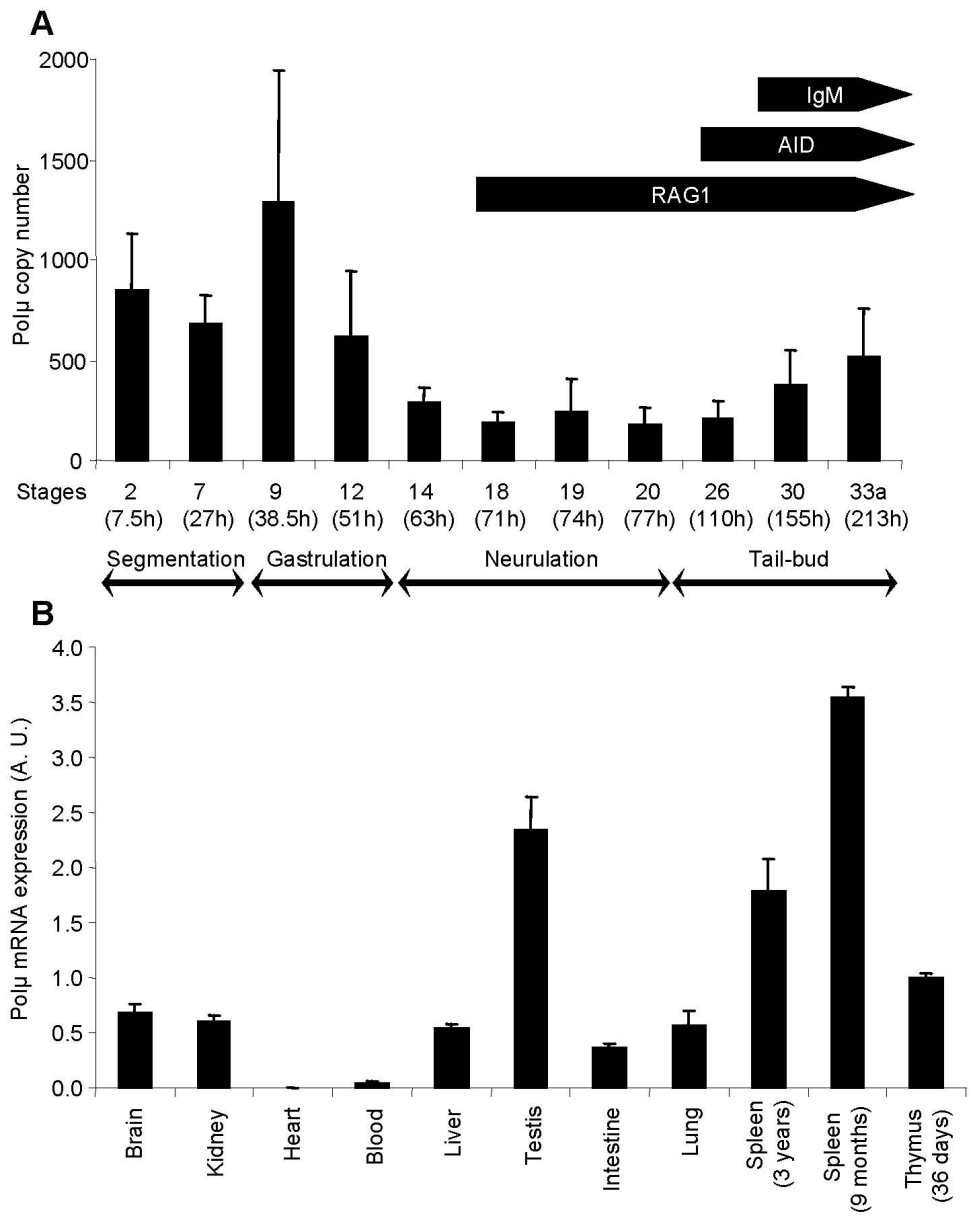
*P. waltl* polymerase µ mRNA expression profiles. Polµ transcripts were quantified by real-time PCR during early stages of development (**A**) and in various tissues (**B**). Arrows indicate the expression of AID, IgM heavy chains and RAG1 transcripts, as reported in previous studies [4], [6]. Developmental stages, as well as the time elapsed since laying (between brackets), are indicated. Ten embryos were studied for each developmental stage. The relative value obtained for the thymus of stage 44 larvae was set to 1 as a reference for tissue expression. Values represent means ± SEM.

To determine when Polµ mRNA is expressed during early *P. waltl* development and situate its expression relative to the first expression of AID, RAG1 and IgM heavy chain transcripts [4], [6], we performed real-time PCR experiments on cDNAs prepared with total RNA extracted from embryos from stage 2 (four cells, 7.5 h after laying) to stage 33a (213 h after laying) of development. Our results (Figure 2A) indicate that the amount of Polµ transcripts is important during the first stages of development, is weak during neurulation and increases again during the tail-bud stages when the first IgM heavy chain mRNAs appear. We also quantified Polµ transcripts in adult tissues, in which strong signals were observed in the testis, the thymus and the spleen (Figure 2B).

### Polµ Expression in Larvae Submitted to Spaceflight and Spaceflight-associated Environmental Conditions

**Figure 3:**
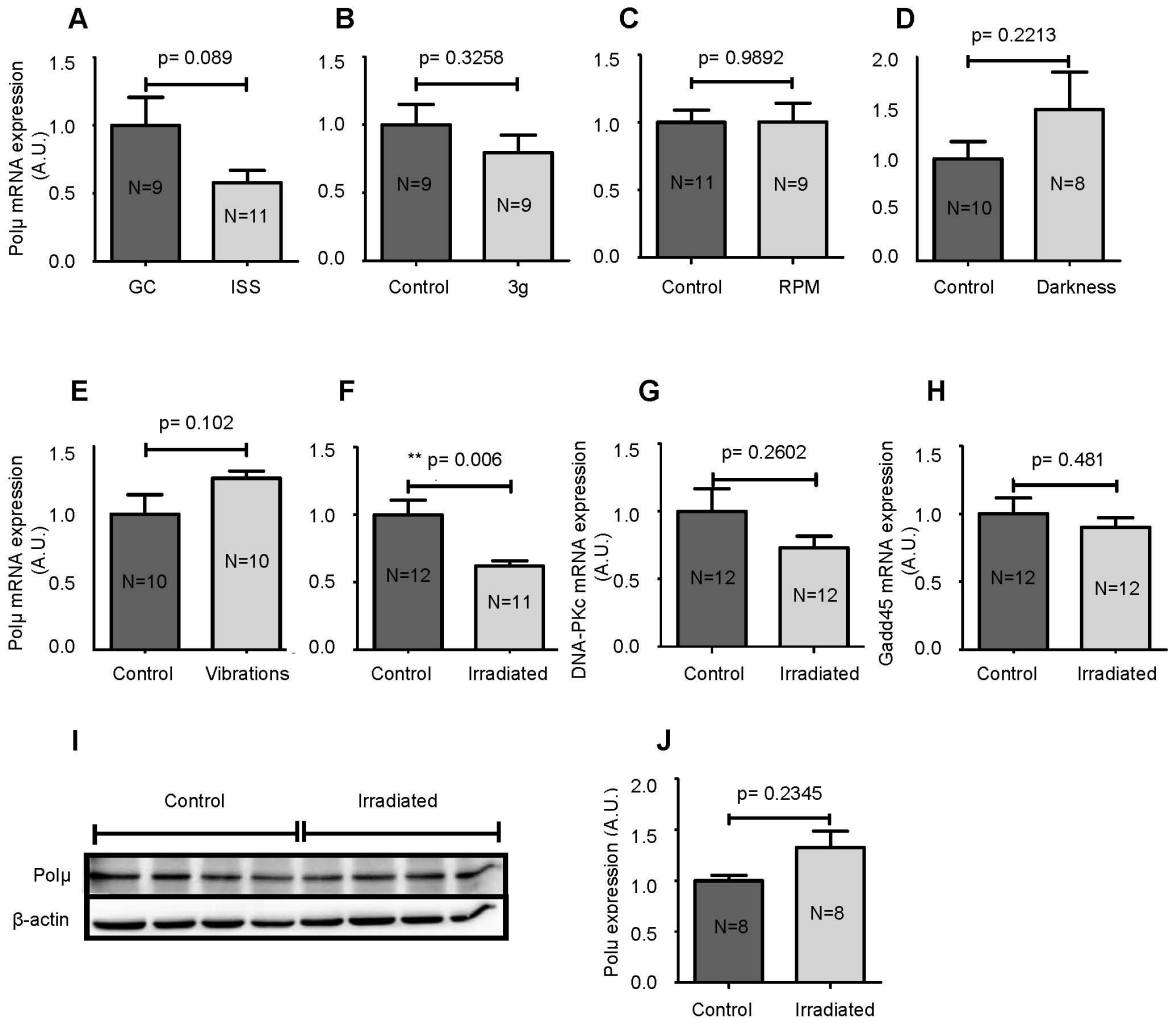
Effects of spaceflight conditions. Polµ mRNAs were quantified in larvae that developed in microgravity in the ISS (**A**), in hypergravity (**B**), in the RPM to simulate microgravity (**C**) or in the dark (**D**). The same study was performed with larvae subjected to a mechanical stress (**E**) or the ISS radiation environment (**F**). The effects of space radiation on DNA-PKc (**G**) and Gadd45 (**H**) mRNAs and on the amount of Polµ protein (**I** and **J**) were also investigated. Western blot results were analyzed and quantified by densitometry using a Fusion Fx7 imaging system (Vilber Lourmat, Marne-la-Vallée, France) and Polµ expression was normalized to β-actin (**J**). Each lane of the western blot contains proteins from 3 larvae. Results are expressed as the mean ± SEM. “A.U.” stands for “arbitrary units”.

Because our previous studies suggested that spaceflight conditions could alter the V(D)J recombination process [8], we quantified Polµ transcripts in larvae of the AMPHIBODY space experiment. Our results show a tendency for a decrease in Polµ transcripts (p = 0.089) in larvae that developed in the ISS compared to ground controls (Figure 3A). To identify the cause of decreased Polµ transcription, we recreated in the laboratory, to the best of our abilities, the various environmental changes encountered during spaceflight and quantified Polµ mRNAs in larvae subjected to each of these changes (Figures 3B–F). Our results demonstrate that neither hypergravity nor simulated microgravity in the RPM affected the expression of Polµ transcripts, indicating that an alteration in gravity is not responsible for the decrease in Polµ transcription. Perturbation of the circadian rhythm (Figure 3D) and mechanical stress (Figure 3E) also did not affect Polµ transcription, while exposure to simulated space radiation led to a significant decrease in Polµ mRNAs (p = 0.006) (Figure 3F).

Consequently, we wondered whether the simulation of space radiation could also impact the expression of another gene involved in NHEJ, DNA-PKc, a kinase involved in the repair of DNA breaks generated by radiation and Ig gene rearrangement. Our results indicate that space radiation does not affect the transcription of DNA-PKc (Figure 3G). To determine whether the stress created by the irradiation protocol was high, we quantified Gadd45, a canonical stress response gene. As shown in Figure 3H, the transcription of Gadd45 is also unaffected by the simulation of space radiation. Finally, we quantified Polµ protein in control and irradiated larvae. Our results show that space radiation has no impact on Polµ protein levels (Figures 3I and 3J). We attempted to quantify DNA-PKc and Gadd45 protein levels, but the commercial antibodies tested did not recognize these proteins in *P. waltl* samples.

### Cumulative Effects of Space Radiation and Circadian Rhythm Perturbation

**Figure 4:**
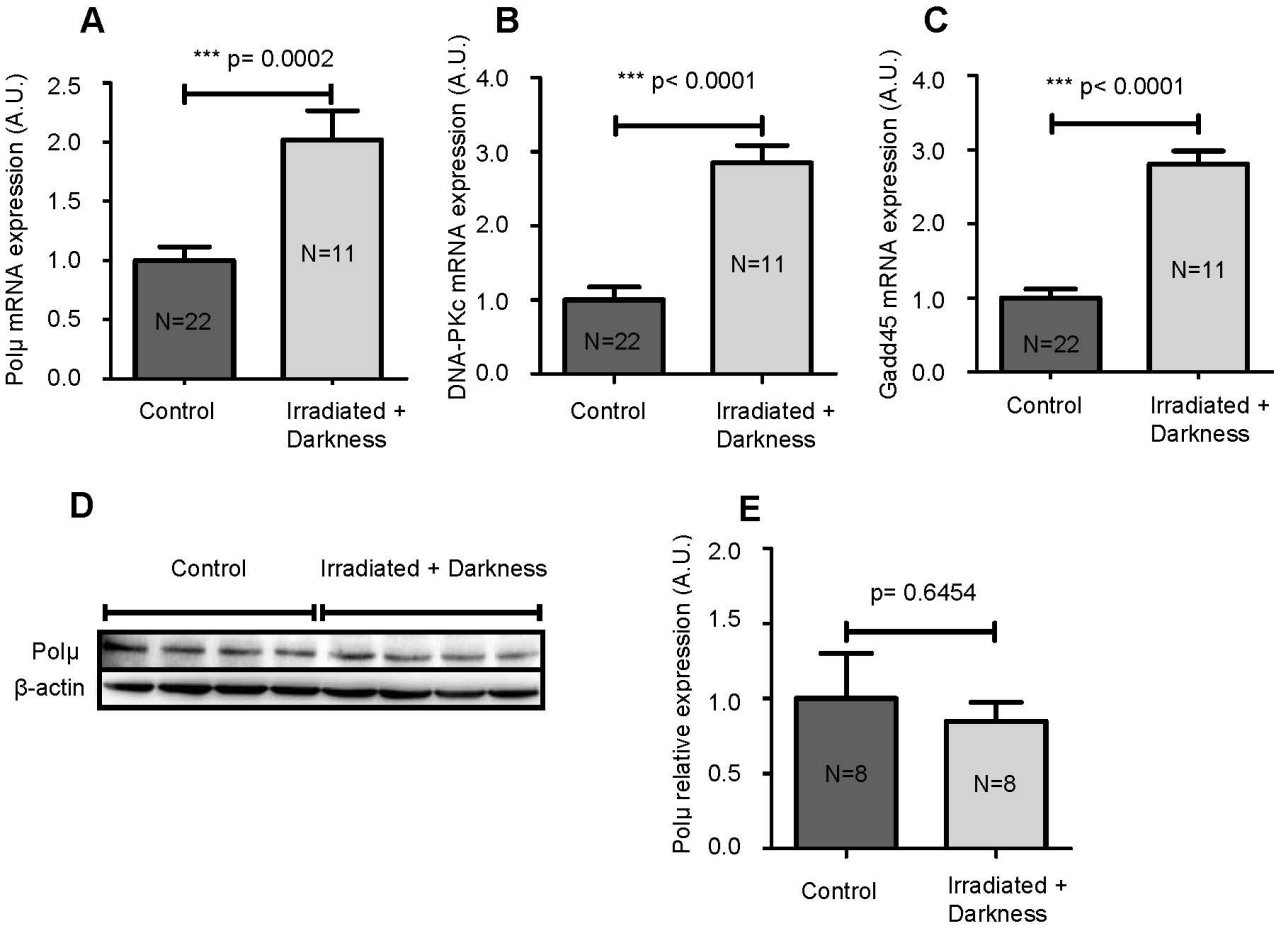
Effects of the combination of space radiation and darkness. Polµ (**A**), DNA-PKc (**B**) and Gadd45 (**C**) mRNAs were quantified by qPCR in larvae that developed in the dark and under the simulation of ISS radiation. Polµ proteins were quantified by western blotting (**D**). Each lane contains proteins from 3 larvae. Western blot results were analyzed and quantified by densitometry using a Fusion Fx7 imaging system (Vilber Lourmat, Marne-la-Vallée, France) and Polµ expression was normalized to β-actin (**E**). Results are expressed as the mean ± SEM. “A.U.” stands for “arbitrary units”.

During spaceflight, astronauts are submitted to a combination of stresses and not to a single stress, as tested above. Therefore, we analyzed the effects of a combination of two environmental modifications on Polµ, DNA-PKc and Gadd45 mRNA levels and on Polµ protein levels. Because it is not possible to test all possible combinations, we opted for the combination of space radiation with the perturbation of the circadian rhythm, as the latter is known to modulate immunity [35]. Exposure of larvae to darkness and simulated space radiation led to significantly increased Polµ mRNA levels (2 fold increase) (Figure 4A) and to the almost tripled expression of both DNA-PKc and Gadd45 transcripts (Figures 4B and 4C). However, the amount of Polµ protein was unaffected by this combination of environmental changes (Figures 4D and 4E). Thus, it appears that space radiation combined with darkness induces a greater response in larvae compared to either condition alone, as shown by increased DNA-PKc, Gadd45 and Polµ mRNA levels, even though the induction of Polµ transcription is not sufficient to alter the amount of Polµ protein.

### Oxidative Stress in Irradiated Larvae and in Larvae Irradiated in the Dark

**Figure 5:**
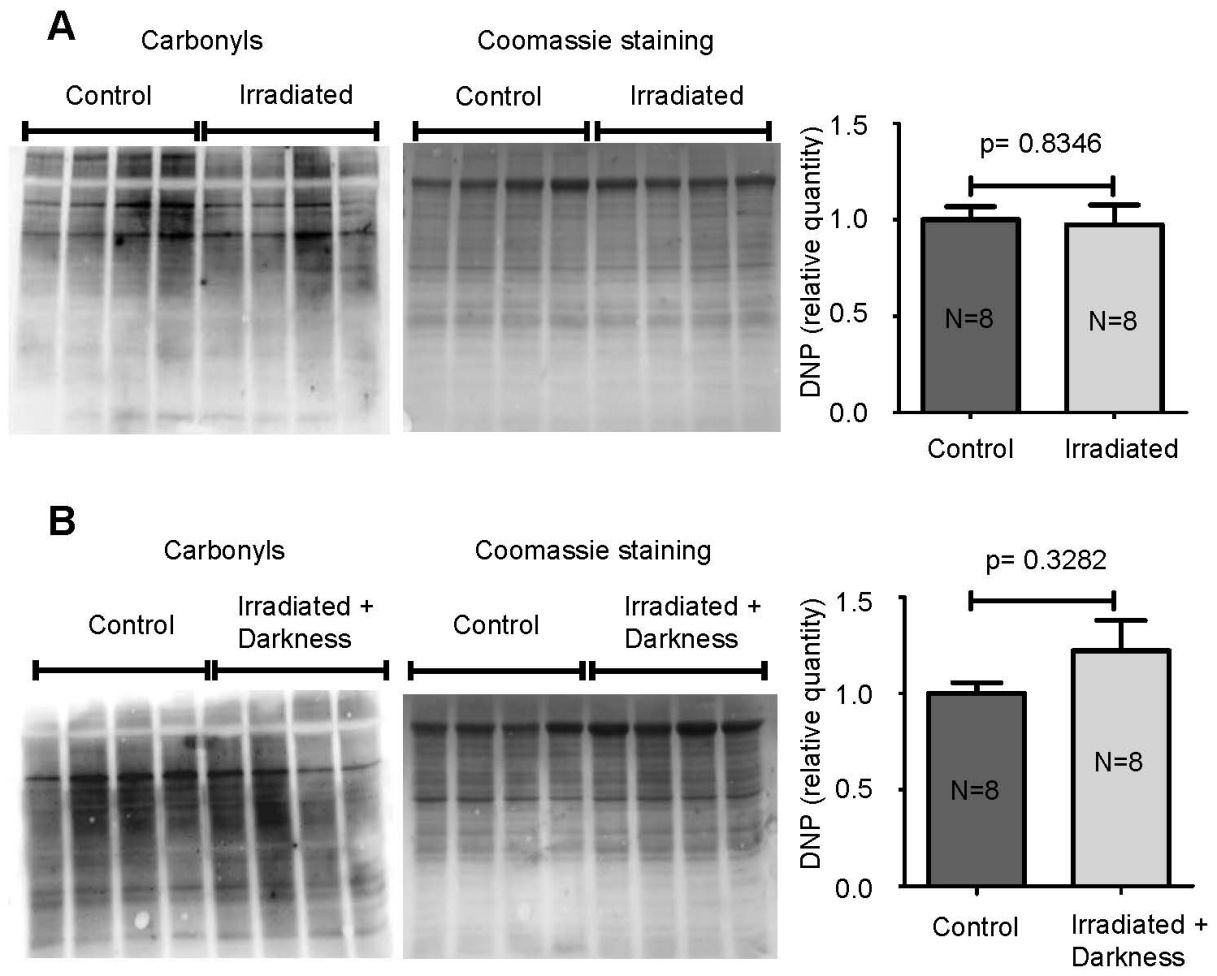
Protein oxidation evaluated by measuring the carbonylation of amino acid side chains. Representative oxiblots of carbonylated proteins from larvae submitted to space radiation (**A**) or larvae that developed in the dark and under the simulation of ISS radiation (**B**) are shown. Each lane contains proteins from 3 larvae. Equal protein loading was assessed by Coomassie staining. Western blot results were analyzed and quantified by densitometry using a Fusion Fx7 imaging system (Vilber Lourmat, Marne-la-Vallée, France) and carbonylation was normalized to the total amount of protein. Results are expressed as the mean ± SEM.

It has previously been shown that radiation can induce oxidative stress and that some markers, such as Gadd45, are sensitive to an oxidative environment. Therefore, we determined whether the variations in mRNA expression found in this study could be due to oxidative stress. To test this hypothesis, we measured the carbonylation of proteins in control and irradiated larvae and in larvae irradiated in the dark. Our results show that neither radiation (Figure 5A) nor the combination of space radiation and darkness (Figure 5B) induced oxidative stress. Thus, the variations of Polµ, DNA-PKc and Gadd45 transcripts described in this study (Figure 4) are not due to increased oxidative stress.

## Discussion

### Overexpression of Full-length Polµ mRNA in *P. waltl*

In this study, we report the first sequencing of Polµ in a urodele amphibian. We also describe a splice variant with a 103 bp deletion, corresponding to exon 8 (Figure 1A), which represents, respectively, 16% and 6% of the 11-exon isoform expressed in the spleen and testis of adults (Figure 1B). No other splice variant was revealed by our RACE-PCRs. Numerous human and murine Polµ splice variants have been described representing approximately 90% of Polµ mRNA species, in which splicing affected exons 5 to 11, encoding the active site of the polymerase. In most cases, these splicing events induced a premature stop codon and truncated proteins [23], [27], which could function as regulators of the activity of this enzyme [36]. Similarly, the splice variant detected in this study lacks one part of its active site and will consequently likely be nonfunctional. However, in *P. waltl*, the full-length Polµ isoform represents 84–94% of Polµ mRNAs. This corresponds to an 8–9 fold overexpression compared to humans and mice, in which the full-length isoform represents only ∼10% of the total Polµ mRNAs. Given that the overexpression of full-length Polµ in the RAMOS cell line increased the frequency of somatic hypermutations at G/C residues and decreased those at A/T residues by a factor of 6 [24], this overexpression of full-length Polµ in *P. waltl* could explain why SHM affects mainly G/C residues in Ig VH regions of this animal [11].

### Polµ Expression Profiles

Using real-time PCR, we detected high quantities of Polµ transcripts at early developmental stages (segmentation and gastrulation) (Figure 2A). Expression of Polµ was approximately 4 times lower during neurulation and increased again when Ig heavy chain mRNAs appeared. These results suggest that Polµ has a role during early embryonic development that is distinct from antibody V(D)J recombination. During early ontogeny, somatic cells proliferate actively and are sensitive to genotoxic stresses. Consequently, robust machineries are required to preserve cell viability and genome stability [37], [38]. Polµ, which is a component of the cellular response to DSBs [28], [39], is well-equipped to connect embryonic DSBs and prevent genomic damage, thereby providing an explanation to its very early developmental expression. The fact that Polµ expression increases when Ig heavy chain gene rearrangement starts was expected because it was shown that Polµ adds nontemplated nucleotides at D-J_H_ joints in mouse embryos when TdT is not yet expressed [40].

As reported in humans [23], Polµ is expressed in a wide range of *P. waltl* tissues, with greater representation in lymphoid organs (Figure 2B). Expression in these tissues was expected because we previously detected a high level of RAG1 mRNA in the thymus of 40-day-old larvae and lower levels in the spleens of 2-year-old adults [4], and Polµ repairs RAG-induced DSBs [40]. Figure 2B also shows that the expression of Polµ in the spleen decreases with age. Some authors have suggested that due to its mutagenic potential, its preferential expression in secondary lymphoid tissues and its expression in the nucleus of centroblasts from human tonsils, Polµ could play a role in somatic hypermutation of Ig genes [24]–[27]. However, an analysis of Polµ-deficient mice did not support this hypothesis [36], [41]. This discrepancy could be due to redundancy between DNA polymerases involved in the hypermutation process. Nevertheless, if Polµ is involved in the SHM machinery, the decrease in Polµ expression with age suggests that the efficiency of that machinery, which is required for Ab affinity maturation, could decrease with age.

We also detected a high level of Polµ mRNAs in the testis, a tissue in which many repair or translesional DNA polymerases are strongly expressed. A similar observation was made in the ovary and testis of zebrafish [34]. This expression pattern could be related to early embryonic expression. Indeed, as suggested above, Polµ could be involved in the protection of the genome of germinal cells against DSBs and in limiting the transmission of mutations between generations. This hypothesis is reinforced by evidence that suggests a role for Polλ, another PolX family member, in DNA repair synthesis associated with meiosis, as high expression of this protein was observed in pachytene spermatocytes [42].

### Modulation of Polµ Expression by Spaceflight-encountered Environmental Conditions

Because Polµ plays a role in hematopoiesis [40], V(D)J recombination [20] and potentially SHM, and given our previous studies that demonstrated that the space environment affects Ig gene expression and decreases SHM [2], [8], [10]–[12], [43], we investigated the effects of spaceflight conditions on Polµ expression. Our results show that Polµ transcription tends to decrease after 10 days of development under spaceflight conditions (Figure 3A) and that radiation, and not the other environmental modifications encountered during the flight, seem primarily involved in this down-regulation. The fact that radiation caused a decrease in Polµ mRNA is not surprising because Aoufouchi et al. [23] showed that ionizing radiation downregulates Polµ transcription in RAMOS cells. The decrease in Polµ mRNA in response to space radiation was however not confirmed at the protein level (Figures 3I and 3J). In HEK cells, the amount of Polµ protein was shown to be increased by radiation exposure [28]. However, in that study the cells were exposed to 12 Gy of radiation, while in our study *P. waltl* larvae were exposed to total doses of 1.9 mGy of γ rays and 2.0 mGy of neutrons. Differences in the doses of radiation likely explain why in this study no increase in Polµ protein could be detected. Recently, Wyrobek et al. [44] evaluated transcriptional changes across 5 doses of radiation (1–10 cGy, considered to be low doses) and found Gadd45 to be a low dose response gene. However, even the doses considered to be low in that study were higher than the doses used here to mimic space radiation, thereby explaining why no change in Gadd45 mRNA was detected (Figure 3H).

High doses of radiation (>2 Gy) are known to induce the formation of reactive oxygen species (ROS) [45], which cause damage to biological molecules, including DNA and proteins. Recently, Smith et al. [46] showed that a low dose of radiation (417 µGy/h) produced too few ROS to affect cells. This paper supports our data showing the absence of oxidative stress in larvae that received neutrons and γ rays at a rate of 30 µGy/h to mimic radiations received during 10 days in the ISS (Figure 5).

Because astronauts are exposed to a multitude of environmental modifications during spaceflight, we investigated the cumulative effect of space radiation and another important stress encountered during spaceflight, namely, the disruption of the circadian rhythm. Our results show that the quantity of Polµ mRNA doubled in larvae exposed to radiation and darkness (Figure 4A), while radiation alone decreased the production of Polµ transcripts (Figure 3F), and darkness had no influence (Figure 3D). These observations show that cumulating environmental modifications can induce different effects, likely because a combination of two modifications causes greater stress, as revealed by the amounts of DNA-PKc and Gadd45 mRNAs, which were tripled in larvae irradiated in the dark (Figures 4B and 4C). However, once again, changes in Polµ mRNA levels were not confirmed at the protein level (Figures 4D and 4E). This observation, combined with the absence of protein oxidation (Figure 5), indicates that radiation encountered during a 10 day stay in the ISS has a limited impact, even though Polµ transcription appears to be highly radiation-sensitive given its ability to be modified by the very low doses of radiation used in this study. Nevertheless, this conclusion does not exclude the possibilities that *i*) ROS could be produced under these conditions, as melatonin, whose synthesis is induced by darkness, can act as a free radical scavenger [47] and *ii*) exposure of astronauts to low doses of radiation during very long space mission, such as to Mars, could represent a threat.

### Conclusion

In conclusion, this paper examines polymerase µ of a urodele amphibian and the expression of this enzyme in larvae subjected to spaceflight-induced environmental changes. We noted robust expression of Polµ mRNA during early ontogenesis and in the testis, likely to protect rapidly dividing cells and germinal cells from DNA damage. Polµ expression is also high when Ig gene rearrangements occur, confirming its involvement in V(D)J recombination. Polµ transcripts are less abundant under spaceflight conditions because they are sensitive to space radiation. However, space radiation, alone or in combination with darkness, did not affect the synthesis of the corresponding Polµ protein and did not induce oxidative stress, showing the limited impact of radiation encountered during a 10 day stay in the ISS.
